# School Closure and Mitigation of Pandemic (H1N1) 2009, Hong Kong

**DOI:** 10.3201/eid1603.091216

**Published:** 2010-03

**Authors:** Joseph T. Wu, Benjamin J. Cowling, Eric H.Y. Lau, Dennis K.M. Ip, Lai-Ming Ho, Thomas Tsang, Shuk-Kwan Chuang, Pak-Yin Leung, Su-Vui Lo, Shao-Haei Liu, Steven Riley

**Affiliations:** The University of Hong Kong School of Public Health, Hong Kong Special Administrative Region, People’s Republic of China (J.T. Wu, B.J. Cowling, E.H.Y. Lau, D.K.M. Ip, L.-M. Ho, S. Riley); Centre for Health Protection, Hong Kong (T. Tsang, S.-K. Chuang); Hospital Authority, Hong Kong (P.-Y. Leung, S.-V. Lo, S.-H Liu); and Food and Health Bureau, Hong Kong (S.-V. Lo)

**Keywords:** Influenza, pandemic, mitigation, mathematical modeling, viruses, H1N1, Hong Kong, expedited, dispatch

## Abstract

In Hong Kong, kindergartens and primary schools were closed when local transmission of pandemic (H1N1) 2009 was identified. Secondary schools closed for summer vacation shortly afterwards. By fitting a model of reporting and transmission to case data, we estimated that transmission was reduced ≈25% when secondary schools closed.

The emergence and subsequent global spread of pandemic (H1N1) 2009 presents several challenges to health policy makers. Although some countries have substantial antiviral drug stockpiles available for treatment and chemoprophylaxis and vaccines became available toward the end of 2009, nonpharmaceutical interventions remain the primary resource available to most populations to mitigate the impact of pandemic (H1N1) 2009 (*1*). One such nonpharmaceutical intervention is school closure, either reactively following outbreaks or proactively at district or regional levels (*2*,*3*). A recent review has highlighted the lack of consensus over the potential benefits of school closures and the potential economic and social costs (*4*). Although the current pandemic (H1N1) 2009 virus is of moderate severity, data from 2009 provide an ideal opportunity to estimate the effectiveness of interventions against pandemic influenza.

In Hong Kong Special Administrative Region, People’s Republic of China, there was a considerable delay between the first reported imported case on May 1, 2009, and the first reported local case (i.e., not otherwise epidemiologically linked with outside travel, contact with an imported case-patient, or contact with an infected person who had contact with an imported case-patient) was laboratory-confirmed and reported to the government on June 10. During the initial stages of the epidemic, the local government operated under containment phase protocols, in which all confirmed cases were isolated in hospital and their contacts were traced, quarantined in hotels, hospitals, and holiday camps, and provided with antiviral drug prophylaxis. When the first nonimported case was confirmed, the government entered the mitigation phase and announced immediate closure of all primary schools, kindergartens, childcare centers and special schools, initially for 14 days. Closures were subsequently continued until the summer vacation began July 10. Secondary schools generally remained open, while those with >1 confirmed case were immediately closed for 14 days. Some containment-phase policies, including isolation of cases and prophylaxis of contacts, were maintained until June 27. During our study period, patients seeking treatment for suspected influenza at designated fever clinics and public hospital emergency departments were routinely tested, and pandemic (H1N1) 2009 virus infection was a reportable infectious disease.

## The Study

We analyzed epidemiologic data on laboratory-confirmed pandemic (H1N1) 2009 infections collected by the Hong Kong Hospital Authority and Centre for Health Protection (the e-flu database). The epidemic curve of laboratory-confirmed pandemic (H1N1) 2009 cases showed a biphasic pattern, with a small initial peak in reported cases at the end of June followed by a nadir at the beginning of July and rising incidence after that (Figure, panel A).

**Figure:**
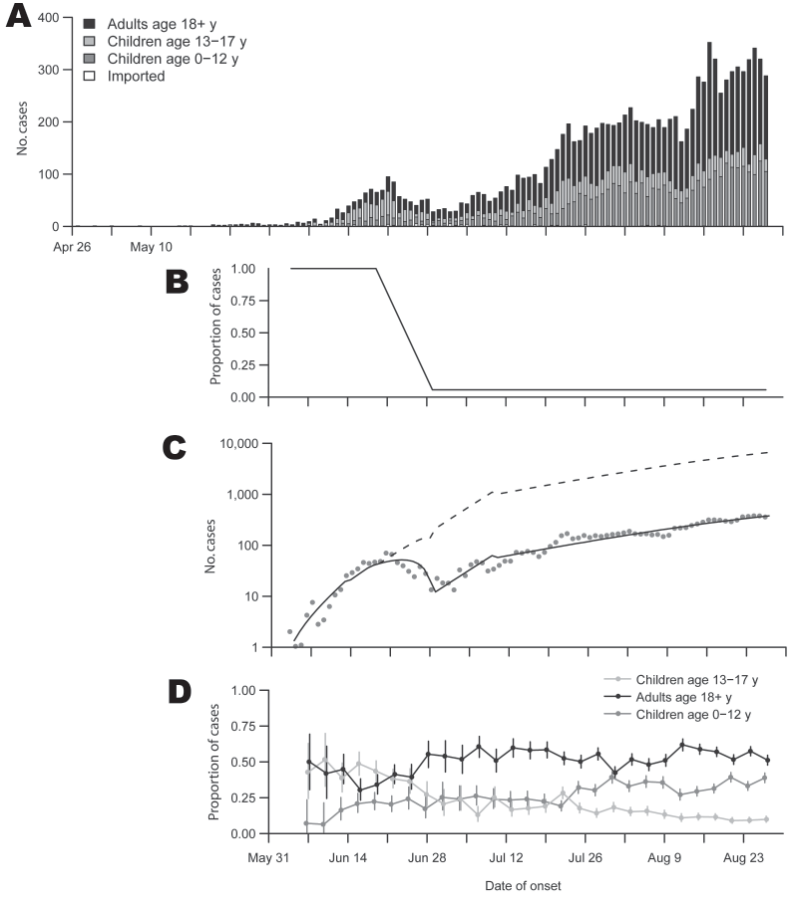
Epidemiologic characteristics of pandemic (H1N1) 2009 in Hong Kong Special Administrative Region, People’s Republic of China, during May through August 2009. A) Time series of laboratory-confirmed pandemic (H1N1) 2009 cases classified as imported or nonimported (by age group) by date of illness onset. B) Estimates of the proportion of cases with illness onset on each day that would subsequently be identified and laboratory confirmed (reporting rates). C) Time series of nonimported pandemic (H1N1) 2009 cases by date of illness onset and the estimates of the underlying true epidemic curve (dashed line) and the fitted observed epidemic curve allowing for changes in reporting rates (solid line). Dots indicate cases reported on a given day. Number of cases plotted logarithmically. D) Distribution of ages of laboratory-confirmed pandemic (H1N1) 2009 cases over time plotted as 3-day rolling averages. Error bars indicate 95% confidence intervals.

We specified an age-structured susceptible-infectious-recovered transmission model to explain the early pandemic (H1N1) 2009 dynamics in Hong Kong (Technical Appendix). We estimated change points in the proportion of symptomatic infections identified and age-specific rates of seeding of infectious cases from overseas. A simple 3-period model for changes in reporting rates provided a parsimonious fit to the data (Figure, panel B). Reporting rates were defined relative to the initial reporting rate. The comparison between the observed and estimated incidence is shown in the Figure, panel C.

We estimated that the relative rate of reporting declined to ≈5.2% of its initial value from June 29 onward (Table). Persons <19 years of age were estimated to be 2.6× more susceptible than the rest of the population. The estimated effective reproductive number was 1.7 before educational institutions for children <13 years of age were closed on June 11, 1.5 between June 11 and July 10 when summer vacation began, and 1.1 for the rest of the summer. The drop in reproductive number was driven by an estimated 70% reduction in intra–age-group transmission concurrent with school closures. The fitted model implies that ≈182,000 persons (2.5% of the population) had experienced illness associated with pandemic (H1N1) 2009 infection by August 27.

**Table:** Summary statistics of posterior distributions obtained by using Markov Chain Monte Carlo in modeling the effects of school closures on mitigating a pandemic (H1N1) 2009 outbreak, Hong Kong, 2009*

Parameter†	Posterior mean (SD)	95% CI
*M_i_*, daily number of effective seeds in age class *i*, *I* = 1,2,3	<13 y: 0.1 (0.1)	0–0.04
	13–19 y: 0.4 (0.1)	0.2–0.6
	>19 y: 0.2 (0.2)	0–0.6
Basic reproductive number	Before Jun 11: 1.71 (0.04)	1.63–1.78
Relative susceptibility of persons <20 y of age	2.64 (0.08)	2.48–2.78
Percentage reduction in intra-age-group transmission given by school closures	70% (3%)	64%–75%
*t_1_*, the date at which reporting rates began to decline	Jun 18 (1.2 d)	Jun 17–Jun 21
*t_2_*, the date at which reporting rates stopped declining	Jun 29 (0.3 d)	Jun 29–Jun 30
*r_2_*, the reporting rate after *t_2_*	5.2% (1.1%)	3.5%–7.7%

*CI, confidence interval. †Model assumes a linear decline in reporting rates from 100% to *r_2_* between times *t_1_* and *t_2_*.

Figure, panel D shows that in the period from the first confirmed local case to the start of summer vacation on July 10, there were a substantial number of cases among older children (whose schools remained open) but few among younger children (whose schools were closed during this period). Only 10% of Hong Kong residents are young children <12 years of age, 8% are older children 13–18 years of age, and 82% are adults.

## Conclusions

In Hong Kong, kindergartens and primary schools were closed when local transmission of pandemic influenza was identified. By using a parsimonious transmission model to interpret age-specific reporting data, we concluded that the subsequent closure of secondary schools for the summer vacation was associated with substantially lower transmission across age groups. We estimated that reporting of cases declined to 5.2% of its initial rate through the second half of June; this is plausible given the gradual change from containment phase to mitigation phase over that period.

It is challenging to infer the precise impact of school closures in Hong Kong, given that they were implemented immediately and sustained until summer vacation and so we have little data on local transmissibility in the absence of school closures. In previous pandemics attack rates have generally been highest in younger children (*4*,*5*), and this has been noted for pandemic (H1N1) 2009 in Mexico (*6*) and Chicago (*7*). This observation, in combination with our finding that children <12 years of age were relatively unaffected in Hong Kong during the school closure period (Figure, panel D), intuitively implies that closures were effective in preventing infections in this age group. Furthermore, assuming that children are responsible for up to half of all community transmission (*8*), it is likely that protection of younger children had substantial indirect benefits. Previous studies have suggested that sustained school closures during a pandemic could reduce peak attack rates and prevent 13%–17% of total cases in France (*8*) or <20% of total cases in the United Kingdom (*3*). Our finding that the reproductive number declined from 1.5 during the kindergarten and primary school closures to 1.1 during summer vacation suggests that a much more substantial drop in attack rates would result from sustained school closures.

By including a model of reporting, we have also been able to estimate case numbers. We estimated a cumulative illness attack rate of ≈182,000 cases (2.5% of the population) by August 27. Between June 29 and August 27, a total of 1,522/9,846 confirmed pandemic (H1N1) 2009 case-patients were hospitalized for medical reasons, among whom 13 died. These numbers are more consistent with a substantially lower case-fatality ratio than suggested by initial estimates of the severity of the pandemic (H1N1) 2009 strain (*9*,*10*). These estimates are dependent on the initial rate of reporting being close to 100%.

We assumed that transmission varied by age and time. If reporting rates varied in a way not accounted for by our model, this would affect the accuracy of our estimates of growth rate and cumulative attack rates. Although we attributed changes in transmissibility between June and August to school closures and summer vacations, it is possible that other secular changes or external factors such as seasonality also contributed. However, it is unlikely that seasonal factors would have reduced transmission of influenza at this time of year, on the basis of symptomatic and laboratory confirmed incidence of influenza from previous years (*11*). Reference data on age-specific population attack rates from serologic surveys or population-based surveillance systems would enable us to calibrate our estimates of reporting rates and growth rates and provide external validation of our model estimates.

## Supplementary Material

Technical Appendix Technical Details of the Transmission Model and Sensitivity Analyses.
