# Supplementary material for: School Closure and Mitigation of Pandemic (H1N1) 2009, Hong Kong
Source: Emerg Infect Dis. 2010 Mar;16(3):538–41. doi: 10.3201/eid1603.091216 (PMC3206396; doi:10.3201/eid1603.091216)
Supplement: Technical Appendix — Technical Details of the Transmission Model and Sensitivity Analyses. [file 09-1216-Techapp.pdf]

School Closure and Mitigation of Pandemic (H1N1) 2009, Hong Kong

Technical Appendix

Technical Details of the Transmission Model and Sensitivity Analyses

Transmission Model

We specified an age-structured Susceptible-Infectious-Recovered model with 3 age classes (<13 years old, 13–19 years old, and >19 years old) to describe the transmission dynamics of influenza A pandemic (H1N1) 2009 in Hong Kong Special Administrative Region, People's Republic of China. The local epidemic was seeded by sporadic imported cases since April 2009. We modeled the seeding force of infection by assuming an effective seed size of M_i per day for class i starting 3 days before the first known symptom onset date of a nonimported case on June 4. The who-acquired-infection-from-whom (WAIFW) matrix W was constructed as follows. First, using recently published social contact data (*1*), we constructed a contact matrix C = $\{C_{ij}\}$ where C_{ij} was the average number of class i contacts that class j individual had per day. The WAIFW matrix W was then constructed from C by 1) assuming that age classes 1 and 2 were h times more susceptible than class 3 (*2*), and 2) scaling the resulting matrix such that the largest eigenvalue of the corresponding next generation matrix was equal to R_0.

We made the following assumptions regarding temporal changes in transmissibility of the virus. Transmissibility remained constant before June 11, the date on which the government announced immediate closure of all primary schools, kindergartens, child-care centers and special schools. Starting on June 11, transmissibility within age class 1 was reduced by a proportion q because of this intervention (i.e., W_{11} was discounted by a factor $1 - q$). Similarly, beginning on July 10 (the start of summer holidays for secondary schools), transmissibility within age class 2 was reduced by the same proportion q. Transmissibility of the virus then remained constant throughout the summer. In summary, the epidemic was simulated by using the following differential equations. For i = 1, 2, 3,

$$\frac{dS_i(t)}{dt} = -S_i(t)\sum_{j=1}^{3} W_{ij}(t)\frac{I_j(t)}{N_i},$$

$$\frac{dI_i(t)}{dt} = -\frac{dS_i(t)}{dt} - \frac{I_i(t)}{D_I} + M_i,$$

where N_i was the size of age class i, $S_i(t)$ and $I_i(t)$ were the number of susceptible and infectious class i persons at time t, $\{W_{ij}(t)\}$ was the WAIFW matrix (time-dependent as described above), and D_I was the mean infectious duration. We assumed $D_I = 3$ days, but our results were insensitive to this assumption (see Sensitivity Analyses below). The parameters R_0, M_i, h and q were estimated by using Markov Chain Monte Carlo (MCMC) methods under a Bayesian inferential framework.

Reporting Rate

We assumed that the proportion of case-patients who reported symptoms to the health officials was described by the following function:

$$r(t) = \begin{cases} 1 & \text{if } t < t_1, \\ 1 + \dfrac{r_2 - 1}{t_2 - t_1}(t - t_1) & \text{if } t_1 \le t \le t_2, \\ r_2 & \text{if } t > t_2, \end{cases}$$

where $0 < r_2 < 1$. That is, we assumed 100% reporting rate before time t_1. The parameters t_1, t_2, and r_2 were estimated by using MCMC (see below).

Statistical Inference

We estimated model parameters based on the time series data of the daily number of confirmed nonimported cases with symptom onset from June 4 through August 27. To this end, we first constructed a likelihood function from the transmission model and the reporting rate model:

$$L(M_1, M_2, M_3, R_0, h, q, t_1, t_2, r_2) = \left(\prod_{i=1}^{3} \prod_{t=1}^{t_A} \frac{(\lambda_{it})^{n_{it}} e^{-\lambda_{it}}}{n_{it}!} \right) \prod_{t=t_A+1}^{t_{\max}} \frac{(\lambda_t)^{n_t} e^{-\lambda_t}}{n_t!}$$

where t_A was the number of days for which age-specific onset data were available (from Jun 4 to Aug 10), λ_{it} and n_{it} were the expected and observed number of onsets in age class i on day t, λ_t and n_t were the expected and observed total number of onsets on day t.

The dataset included 9,918 confirmed cases. The date of symptom onset was available for 4,777 cases. To handle the missing data, we first obtained the empirical cumulative density function (cdf) F_i for the age-specific time delay (x_i) between symptom onset and case confirmation for these 4,777 cases. Technical Appendix Figure 1 displays the resulting histograms. We then assumed that the delay between symptom onset and confirmation for the remaining 5,141 cases followed the same empirical distributions. Following the EM algorithm, we took expectation of the n_{it}'s and n_t's in the likelihood function with respect to these cdfs for cases whose dates of symptoms onset were missing.

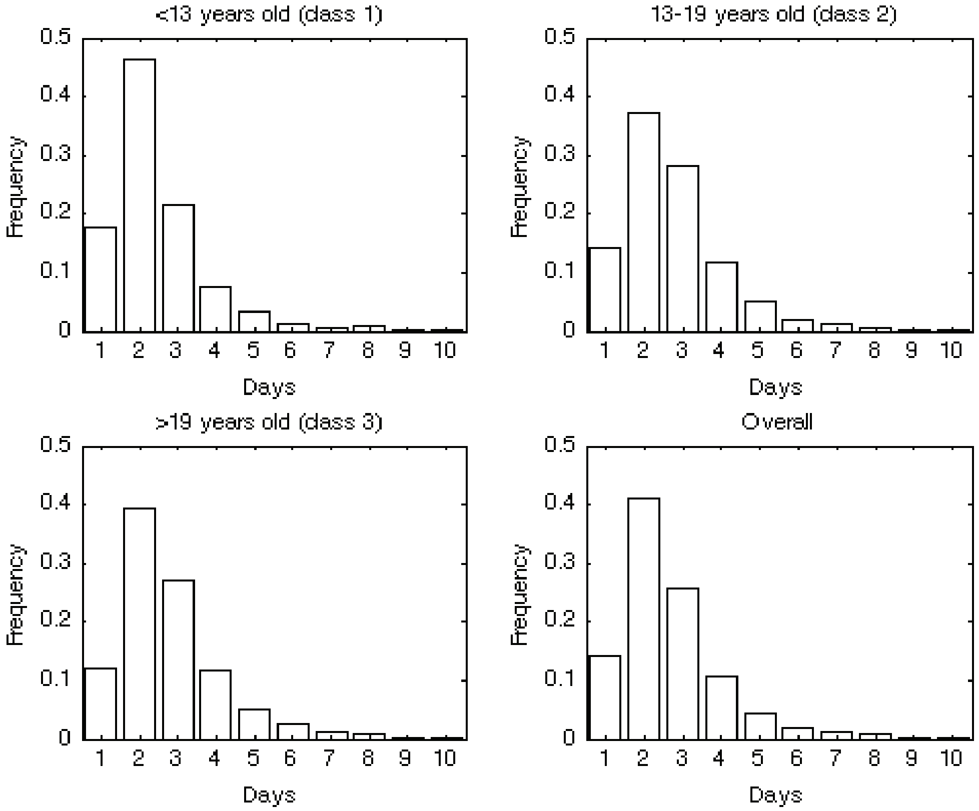

Technical Appendix Figure 1. Histograms for the age-specific and overall time delay between symptoms onset and case confirmation.

Markov Chain Monte Carlo

To simplify the statistical inference procedure and parameter interpretation, we reparameterized the model as follows:

$t_2 = \alpha_2 t_{\max}$,

$t_1 = \alpha_1 t_2 = \alpha_1 \alpha_2 t_{\max}$.

After reparameterization, we assumed flat (i.e., uninformative) prior distributions for all parameters. The prior distributions are given in the Technical Appendix Table.

Technical Appendix Table. Prior distributions and parameter bounds for calculation of posterior distributions using Markov Chain Monte Carlo.

Parameter	Prior	Lower-bound	Upper-bound
M_i, i = 1,2,3	Uniform	0	10
R_0	Uniform	1	2
q	Uniform	0	1
α_1	Uniform	0	1
α_2	Uniform	0	1
r_2	Uniform	0	1
h	Uniform	0	5

To obtain the posterior distributions, we used a random walk metropolis algorithm in which a random step size is chosen for each parameter at every iteration. The step size for a parameter was a uniform random variable with maximum size equal to 1/500 of the feasible range of the parameter (i.e., [upperbound – lowerbound]/500). The MCMC was run for 3,000,000 iterations and the posterior distributions were obtained by using the final 2,000,000 iterations. The acceptance proportion was ≈36%. The results are shown in the Table in the main text and in Appendix Figure 2 below. The posteriors were all distinct from their priors. Chains with different starting points gave similar posterior distributions.

Sensitivity Analyses

We checked the robustness of our results by varying the mean infectious durations D_I from 2 to 4 days. The results were similar to those in the Technical Appendix Table and Technical Appendix Figure 2.

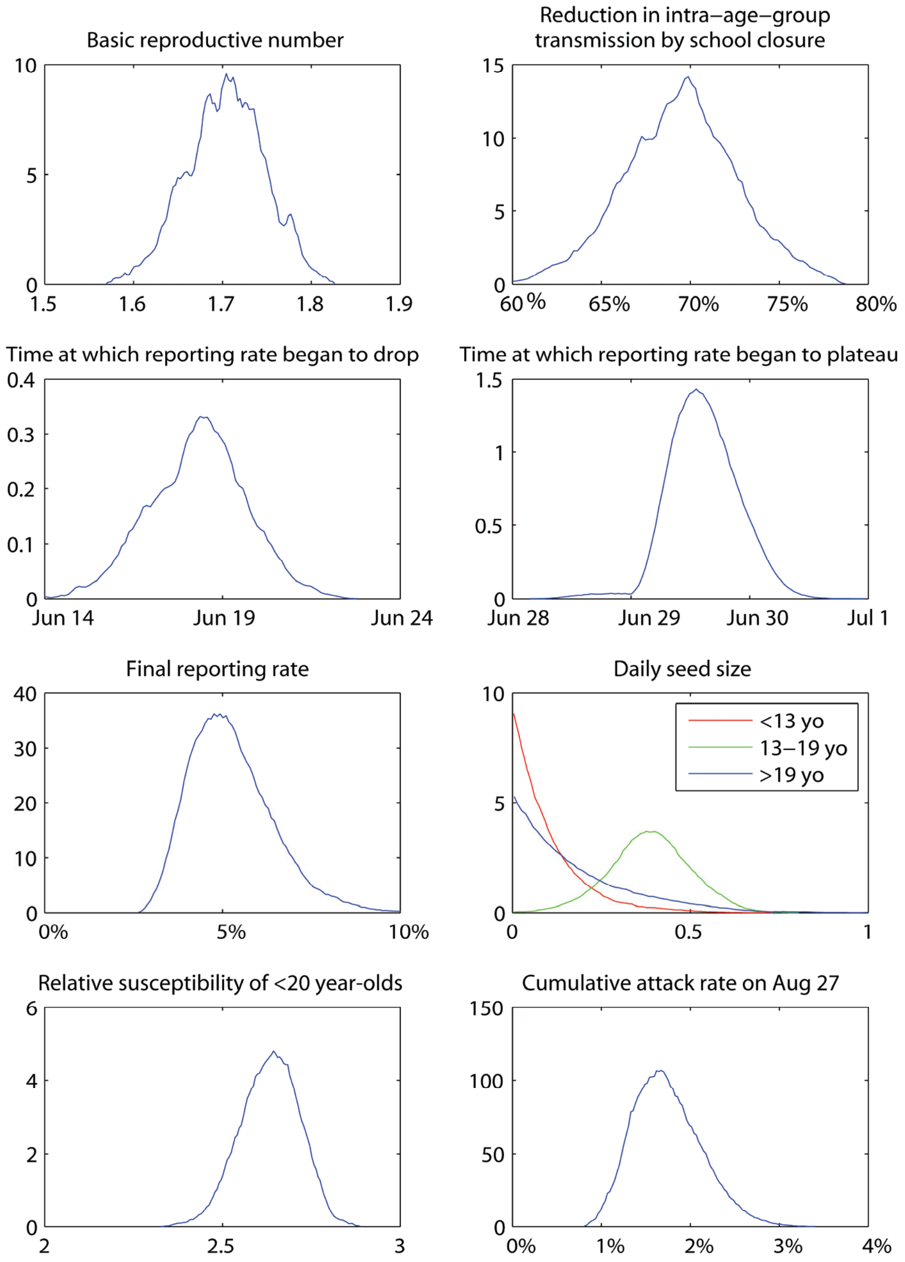

Technical Appendix Figure 2. Posterior distributions of parameters.

References

1. Mossong J, Hens N, Jit M, Beutels P, Auranen K, Mikolajczyk R, et al. Social contacts and mixing patterns relevant to the spread of infectious diseases. PLoS Med. 2008;5:e74. PubMed DOI: 10.1371/journal.pmed.0050074

2. Fraser C, Donnelly CA, Cauchemez S, Hanage WP, Van Kerkhove MD, Hollingsworth TD, et al. Pandemic potential of a strain of influenza A (H1N1): early findings. Science. 2009;324:1557–61. PubMed DOI: 10.1126/science.1176062
